# Built environment and emergency medical services response time in a high-density megacity: multilevel evidence from urban–suburban

**DOI:** 10.3389/fpubh.2026.1930880

**Published:** 2026-10-30

**Authors:** Jingwei Li, Hongjie Wu, Yujie Shen, Ziyue Yu, Hui Chen, Jianghao Yu

**Affiliations:** 1 School of Architecture and Design, Beijing Jiaotong University, Beijing, China; 2 Department of Urban Planning and Design, The University of Hong Kong, Hong Kong, Hong Kong SAR, China; 3 Beijing Emergency Medical Center, Beijing, China; 4 School of Architecture, Tsinghua University, Beijing, China

**Keywords:** built environment, emergency medical services (EMS), multilevel model, response time, urban–suburban heterogeneity

## Abstract

**Introduction:**

Emergency medical services (EMS) response time is a key indicator for evaluating the performance of public health system and is closely associated with patient survival rates. However, empirical evidence on the potential relationship between the built environment and EMS response time remains limited, restricting the development of spatially informed strategies for improving EMS system operations.

**Methods:**

This study employed 2024 ambulance dispatch data from the Beijing Emergency Center Office Management System, combined with multi-source data on the built environment, to construct a multilevel regression model examining the influence of subdistrict (township)-level built environment factors on EMS response time and investigating the heterogeneity between urban areas and suburbs.

**Results:**

The results indicate that, at the citywide level, lower residential facility density and denser arterial and local road networks significantly reduced EMS response time. Specifically, while in central urban areas, high densities of residential facilities were associated with longer response time, in suburban areas, higher densities of local roads enhanced EMS efficiency.

**Discussion:**

These findings provide valuable evidence to guide built environment interventions aimed at shortening EMS response time and improving service efficiency across different areas.

## 1 Introduction

Emergency medical services (EMS) constitute a critical component of healthcare systems, providing timely treatment to patients experiencing accidents, sudden illnesses, or emergencies through call reception, professional intervention, patient transport, and multi-agency coordination, thereby reducing morbidity and mortality (1–3). EMS response time, defined as the interval from the receipt of an emergency call at the dispatch center to the arrival of an ambulance at the scene, is a core metric that directly affects patient survival and treatment outcomes (4–7). In developed countries such as the United Kingdom, the United States, and Germany, target EMS response time for life-threatening emergencies generally range from 7 to 15 min (7–9). Since 2023, average EMS response time in Shanghai, Shenzhen, and Beijing has been reduced to within 12 min (10–12). While approaching international benchmarks, EMS response time still falls short of the U.S. NFPA industry standard of 8 min (8, 13, 14). These gaps highlight the need for targeted strategies to enhance EMS efficiency, ensure timely care for critically patients, and ultimately improve survival outcomes.

Existing research indicates that EMS response time is influenced by multiple factors, including EMS service capacity, traffic conditions, weather, and socioeconomic characteristics. At the service-specific level, optimized EMS station density and vehicle allocation can reduce service radius, shorten EMS response time, and ensure timely care (15, 16). Longer distances from call locations to ambulance stations and higher levels of EMS demand also increase dispatch and EMS response time (17, 18). Traffic congestion is a major contributor to EMS delays, particularly during peak hours when ambulances may be impeded in timely passage through traffic (19–21). Adverse weather, such as rain, snow, dust storms, or extreme heat or cold, can deteriorate road conditions, reduce visibility and mobility, and complicate ambulance deployment, thereby prolonging ambulance travel time (22, 23). Furthermore, studies indicate that EMS response time is longer in low-income areas compared to affluent regions, thus less likely to meet national standards (24).

In addition, the built environment is recognized as a key factor influencing EMS response time (21, 25). Previous research has examined the effects of density, land use, and road network characteristics. Studies on low-density countries such as the United States have shown that urban sprawl, characterized by low building density, poor street connectivity, and limited land-use mix, is associated with longer EMS response time (26, 27). In contrast, research in high-density cities in China and India suggests that districts with highly concentrated populations may experience greater EMS demand, increasing the likelihood of EMS supply shortages and resulting in delayed responses (15, 28). Regarding land use, both the type and spatial configuration of land can affect EMS response time. Medical and commercial land uses are generally associated with shorter EMS response time (16, 25), whereas excessively high residential density may result in prolonged EMS response time (29). Road network accessibility is a critical factor enabling rapid ambulance movement, with higher road density and better connectivity likely to reduce travel time (17, 30). As ambulance travel speeds differ across road types, their influence on EMS response time may vary, although this issue has received limited attention in the literature.

Compared with urban areas, patients in suburban areas typically face longer EMS response time (31–33). For example, the average EMS response time in suburban areas of Denmark is 12.2 min, whereas in urban areas it is only 7.1 min (34). In the United States, a study of cardiac arrest cases found that average EMS response time in urban areas was 1.6 min faster than in suburban areas (35). Some studies have explored urban–suburban differences in EMS response time from the perspective of emergency resources (36–38). Compared with suburban areas, urban regions typically feature denser EMS station layouts, which shorten service distances and improve response efficiency (16). Urban areas also tend to have larger ambulance fleets and highly trained EMS personnel, which further enhances dispatch effectiveness and service quality (39). Nonetheless, substantial differences in road networks, facility layouts, and population distribution between urban and suburban areas may also contribute to unequal EMS response time, the mechanisms underlying which, however, remain insufficiently understood.

Studies examining the relationship between the built environment and EMS response time have commonly employed traditional linear models, including ordinary least squares and linear quantile models (40, 41), spatial econometric models such as spatial lag models, spatial error models, and geographically weighted regression (GWR) (17, 42), and non-linear machine-learning models such as random forests, gradient boosting, and XGBoost (21). While these approaches account for spatial heterogeneity and complex non-linear associations, a critical methodological issue remains: individual EMS response times are hierarchically nested within spatial units (e.g., counties, blocks) and their corresponding built environment contexts. Ignoring this multilevel data structure may lead to ecological fallacies and bias empirical estimates, potentially resulting in misleading policy recommendations.

To address these gaps, our study employed a multilevel multiple regression model to examine the effects of subdistrict (township)-level built environment factors on EMS response time. We focus on two questions: (1) To what extent does the built environment influence EMS response time? (2) How does this influence differ between urban and suburban areas? The analysis is based on 211,685 real ambulance dispatch records from a 4-month period in 2024 in Beijing. Indicators of the subdistrict-level built environment were constructed along three dimensions: density, land use, and road networks. An individual-subdistrict two-level regression model was then used to assess the impact of built environment factors on individual EMS response time and their urban–suburban heterogeneity. This study contributes to understanding how the built environment affects EMS service efficiency in high-density cities, providing evidence-based insights to support healthy city initiatives and sustainable urban development.

## 2 Materials and methods

### 2.1 Research framework

The detailed research framework is illustrated in Figure 1. This study applied a multilevel regression model to examine the effects of the built environment on EMS response time. At the subdistrict level, variables include the built environment, socioeconomic characteristics, and EMS service capacity. At the individual level, variables capture factors specific to each emergency event, such as peak-period dispatch, adverse weather, and EMS distance. Built environment factors, including density, land use, and road network indicators, serve as the key independent variables, while the remaining factors are control variables. To investigate urban–suburban heterogeneity in the impact of built environment on EMS response time, the analysis constructed models for the full citywide sample and two subsamples representing the central urban and suburban areas.

**Figure 1:**
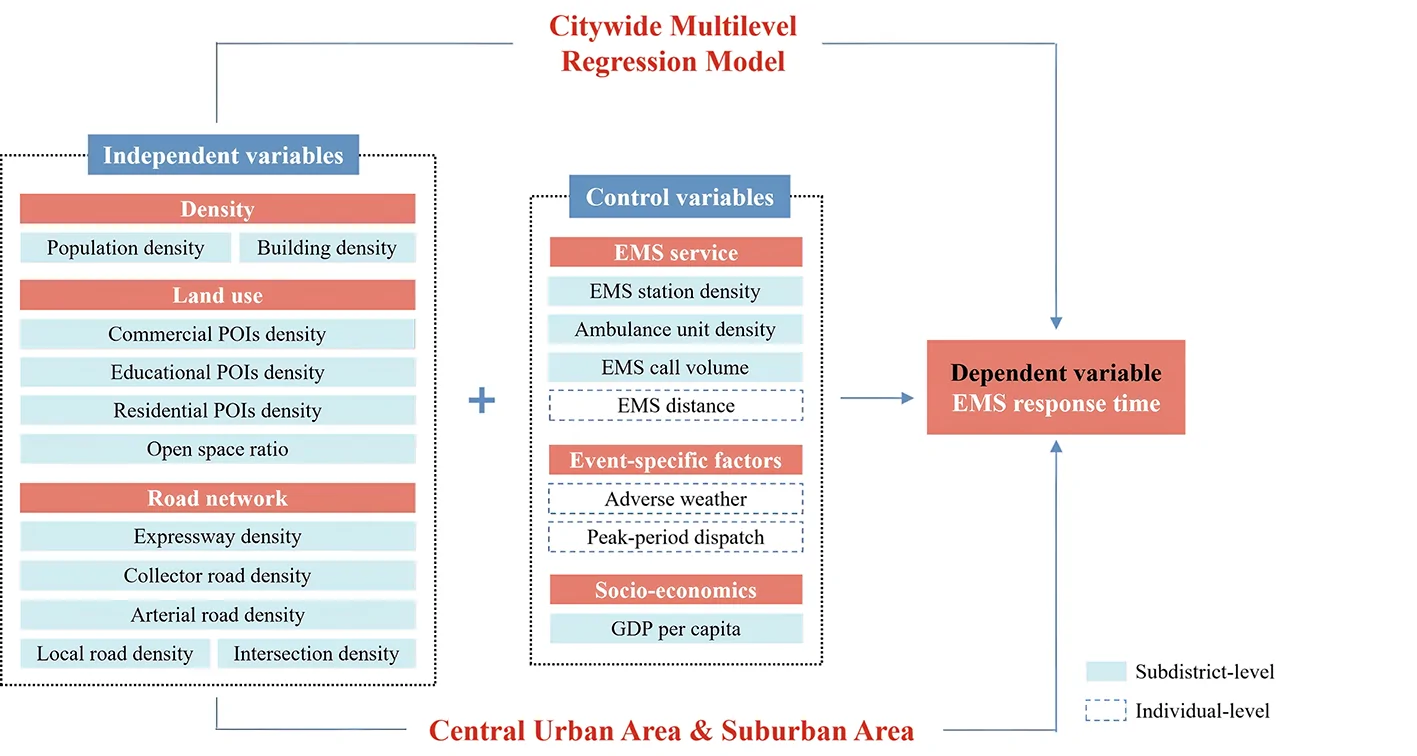
The research framework.

### 2.2 Study area

This study focused on the entire municipality of Beijing, including 16 districts and 334 subdistrict units, with a total area of 16,410 km^2^ and a permanent population of approximately 21.893 million (43). By 2024, Beijing had established 473 ambulance stations, of which 204 are located in the central urban districts (Dongcheng, Xicheng, Haidian, Chaoyang, Fengtai, and Shijingshan) and 269 in the suburban districts (Changping, Daxing, Fangshan, Huairou, Mentougou, Miyun, Pinggu, Shunyi, Tongzhou, Yanqing, and Economic-Technological Development Area) (Figure 2). Beijing's EMS station deployment is planned to ensure at least one ambulance station per subdistrict, with a service target of approximately 10-min driving-time coverage in built-up areas (including peak periods) and a fleet benchmark of roughly one ambulance per 30,000 residents at the district/county level. At the dispatch and operational level, the Beijing 120 emergency dispatch system centrally receives emergency calls, records the patient's condition, location, and contact information, and classifies and manages cases according to standard procedures. After receiving dispatch orders, ambulance teams depart as required and transport patients to appropriate medical institutions based on proximity, urgency, specialty matching, and, when feasible, patient preference (44). In 2024, the average emergency medical service response time in Beijing was 10.04 min (10), sustained efforts are still required to meet the international standard of 8-min EMS response time.

**Figure 2:**
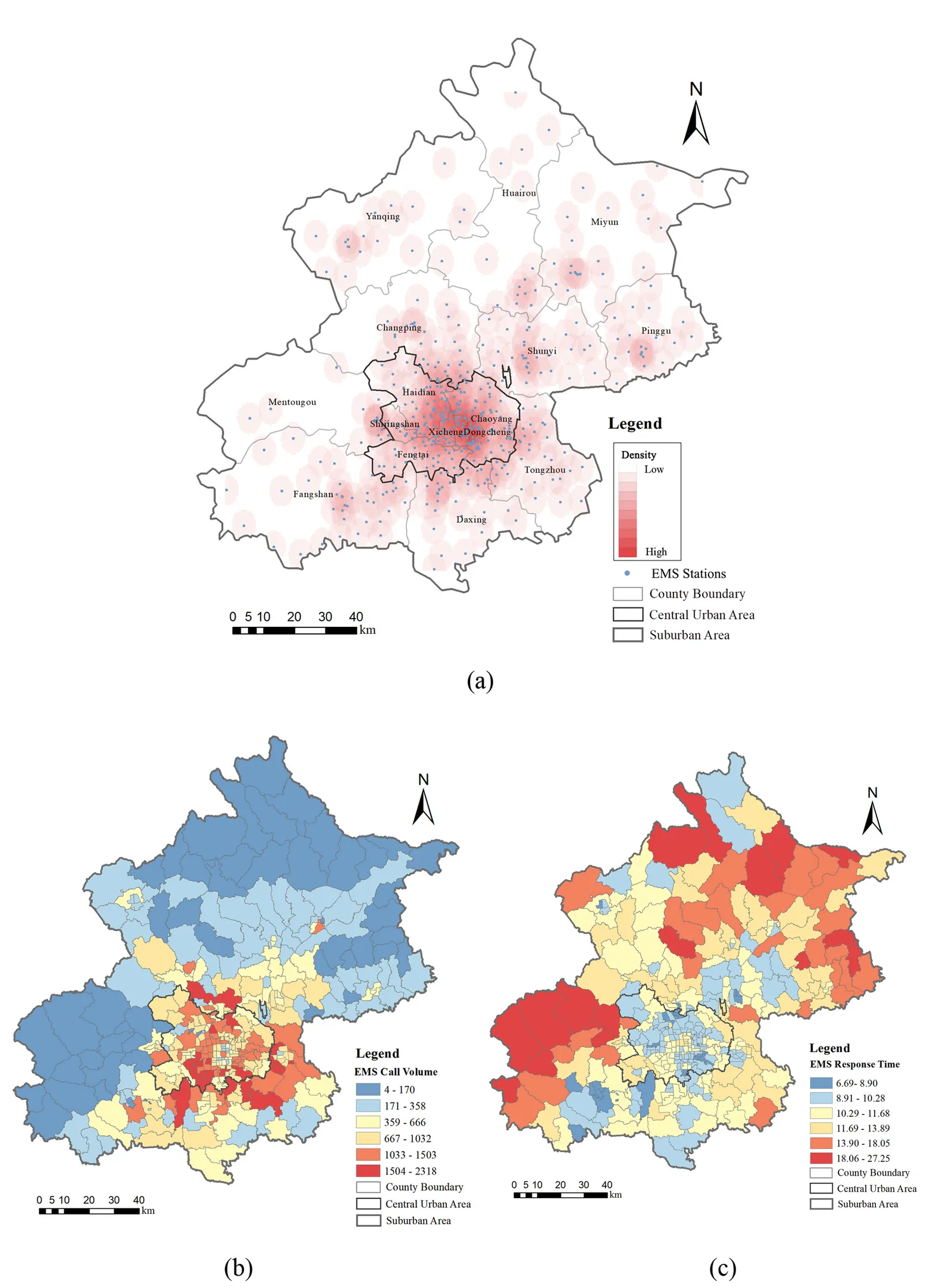
Spatial distributions of EMS facility density, EMS call volume, and EMS response time in Beijing: **(a)** relative density of EMS facilities; **(b)** distribution of EMS call volumes; **(c)** average EMS response time at the subdistrict-level.

### 2.3 Data and measures

#### 2.3.1 Dependent variables

EMS response time was defined as the interval from receipt of an emergency call at the emergency center to ambulance arrival at the scene. This interval encompasses call processing and dispatch, turnout, and travel to the scene (45). The study focused on the overall call-to-scene interval.

EMS response time data were obtained from the Beijing Emergency Center Office Management System. Ambulance dispatch logs from January, April, July, and October 2024 across all 16 districts of Beijing were extracted to provide observations from winter, spring, summer, and autumn, respectively. Only EMS events with a mission type of “treatment” and a status of “completed” were retained. To ensure data quality, records with response times shorter than 1 min or longer than 60 min were excluded as potential outliers (46–49). The final dataset comprised 211,685 EMS events, including 121,751 events in central urban areas and 89,934 events in suburban areas.

#### 2.3.2 Independent variables

Subdistrict-level built environment characteristics were categorized into three dimensions: density, land use, and road networks. Density includes population density and building density, reflecting the concentration of residents and structures. Population data were obtained from the Seventh National Population Census of China (70), and building data were sourced from Baidu Maps (71). Building density was calculated as the ratio of total building footprint area to subdistrict area. Land use encompasses densities of commercial, educational, residential facilities, as well as the proportion of open space. POI data were obtained from Amap (Gaode) (72), and facility density was calculated by dividing the total number of each type of facility by the subdistrict area. Road network data were sourced from OpenStreetMap (OSM), including densities of roads at different hierarchical levels (expressways, arterial roads, secondary roads, and local roads) and intersection density. Road density was calculated as the total length of roads at each level divided by subdistrict area, reflecting the provision of transportation infrastructure and the coverage of the road network. Intersections are typically defined as the convergence of two or more roads on the same plane (50), and intersection density was calculated as the number of intersections divided by subdistrict area.

#### 2.3.3 Control variables

Control variables were categorized into three types: EMS service capacity, event-specific factors, and socioeconomic characteristics. EMS service capacity includes EMS station density, ambulance unit density, EMS distance, and EMS call volume, with all data obtained from the Beijing Emergency Center Office Management System. EMS distance refers to the straight-line distance between the emergency call location and the ambulance location and was used as a proxy for spatial proximity (51, 52). EMS call volume represents the total number of calls received within the corresponding time interval for each subdistrict. Event-specific factors include traffic and weather conditions. Ambulance dispatches between 7:00–9:00 and 17:00–20:00 were defined as peak traffic periods (53, 54). Meteorological data were obtained from the National Oceanic and Atmospheric Administration (NOAA). Based on the Beijing Extreme Weather Criteria and 2024 Beijing meteorological warnings, extreme high temperatures (three consecutive days with maximum temperature above 37 °C), extreme low temperatures (minimum temperature below −12 °C), abnormal weather events (e.g., rain, snow, fog, sandstorms, hail), and severe pollution (AQI 201–300) were defined as adverse conditions affecting ambulance mobility (55, 56). Socioeconomic characteristics were measured by per capita GDP at the subdistrict level, with data sourced from the Beijing Statistical Yearbook 2024. The definitions and calculations of the study variables are presented in Table 1.

**Table 1:** Definitions and calculations of the variables.

Category	Variable	Definition and calculation
Density	Population density (people/km^2^)	The total number of people in the subdistrict divided by the total area of the subdistrict
	Building density (%)	The ratio of the total base area of the buildings in the subdistrict to the total area of the subdistrict
Land use	Commercial POIs density (*n*/km^2^) Educational POIs density (*n*/km^2^) Residential POIs density (*n*/km^2^) Open space ratio (%)	The total number of commercial/educational/residential POIs in the subdistrict divided by the total area of the subdistrict Commercial POIs include shopping services, catering services, financial and insurance services, daily services, companies and office buildings Educational POIs include kindergartens, primary schools, secondary schools, colleges, technical schools, and adult education Residential POIs include housing, villa, community center and etc. The ratio of the total area of the open space to the total area of the subdistrict
Road networks	Expressway density (km/km^2^) Arterial road density (km/km^2^) Collector road density (km/km^2^) Local road density (km/km^2^) Intersection density (*n*/km^2^)	The total length of expressway/arterial road/collector road/local road divided by the total area of the subdistrict The total number of intersections in the subdistrict divided by the total area of the subdistrict
EMS service	EMS station density (*n*/10,000 people) Ambulance unit density (*n*/10,000 people) EMS distance (km) EMS call volume (*n*)	The total number of EMS stations/ambulances in the subdistrict divided by the total number of population, multiplied by 10,000 Straight-line distance between the EMS location and the call location The total number of EMS call volume during the receipt time
Event-specific conditions	Adverse weather	0 = normal weather, 1 = adverse weather (extreme temperatures, rain, snow, fog or sand storms, severe air pollution)
	Peak-period dispatch	1 = morning peak (7:00–9:00) and evening peak (17:00–20:00); 2 = non-peak hours.
Socio-economics	GDP per capita	The total GDP of the subdistrict divided by the total number of population

### 2.4 Statistical analysis

The study hypothesized that the built environment may affect EMS response time, and a multilevel multiple regression model was built to explore this relationship in general. Multicollinearity was assessed by calculating the variance inflation factor (VIF), and variables with VIF values below 10 were retained (57, 58).

Given that factors influencing EMS response time operate at both the individual and subdistrict levels, a two-level regression model is statistically appropriate for this analysis (59, 60). Prior to modeling, we estimated a null model and used the intraclass correlation coefficient (ICC) to evaluate the need for a multilevel approach. The model is formulated as follows Equation 1:


$$y_{ij}=\gamma_{00}+\gamma_{01}w_{j}+\beta_{1}x_{ij}+\mu_{0j}+\varepsilon_{ij}$$ (1)

where, *y*_*ij*_ represents the EMS response time, γ_00_ denotes the overall average intercept, γ_01_ indicates the average effect of subdistrict-level built environment variables *w*_*j*_ on EMS response time, β_1_ represents the fixed effects of individual-level variables *x*_*ij*_, and μ_0*j*_+ε_*ij*_ corresponds to the random effects component.

## 3 Results

### 3.1 Spatial patterns of EMS response time

Figures 2a–c show the spatial distribution of EMS facilities density, EMS call volume, and EMS response time in Beijing, respectively. The distribution density of EMS facilities in Beijing exhibits a characteristic pattern of decreasing from the urban center toward the periphery. Central urban districts such as Dongcheng, Xicheng, Chaoyang, and Haidian have formed a relatively dense emergency service network with a high concentration of stations. In contrast, outer suburban districts including Mentougou, Miyun, Huairou, and Yanqing are characterized by generally sparse coverage, with only localized clusters observed in certain areas, mostly concentrated around district-level central towns.

During the study period, EMS call demand also exhibited significant spatial clustering. High call volumes were mainly concentrated in the central urban area and suburban transition zone, forming a contiguous high-demand region. The highest call volume was recorded in Yongshun subdistrict, reaching 2,318 calls. In contrast, most subdistricts in the outer suburbs fell into the low call volume category, with high values only emerging around a few peripheral new towns or district centers, for instance, Beiqijia subdistrict with 2,112 calls. Overall, the call volume in the central urban area was 1.4 times that of the suburban area.

The average EMS response time in Beijing was 10.54 min, showing distinct spatial heterogeneity that gradually increased from the urban center to the surrounding areas. The average EMS response time in the central urban area was 10.16 min, where most subdistricts belonged to the low response time category, such as Panjiayuan subdistrict (7.77 min) and Dongsheng subdistrict (8.14 min). Although the average EMS response time in suburban areas was 11.04 min, severe response delay zones with EMS response time approaching 30 min existed in the border areas of the outer suburbs, including Yanchi subdistrict (27.25 min) and Qingshui subdistrict (24.42 min).

Figures 3A–C illustrate the distribution differences in EMS response time at the district level. In central urban districts such as Dongcheng, Xicheng, Chaoyang, Haidian, Fengtai and Shijingshan, over 76% of EMS call volumes were answered within 12 min, and Shijingshan performed particularly well, with proportions exceeding 80%. In contrast, only about 70% of EMS call volumes were answered within 12 min in suburban areas, with Pinggu having the lowest proportion at 51.11%. Overall, the number of EMS call volumes within 8 min in central urban districts is approximately 1.5 times that in suburban districts, while EMS call volumes within 8–12 min are about 30% higher. Central urban districts exhibit shorter EMS response time compared to suburban districts (Figures 3A–B).

**Figure 3:**
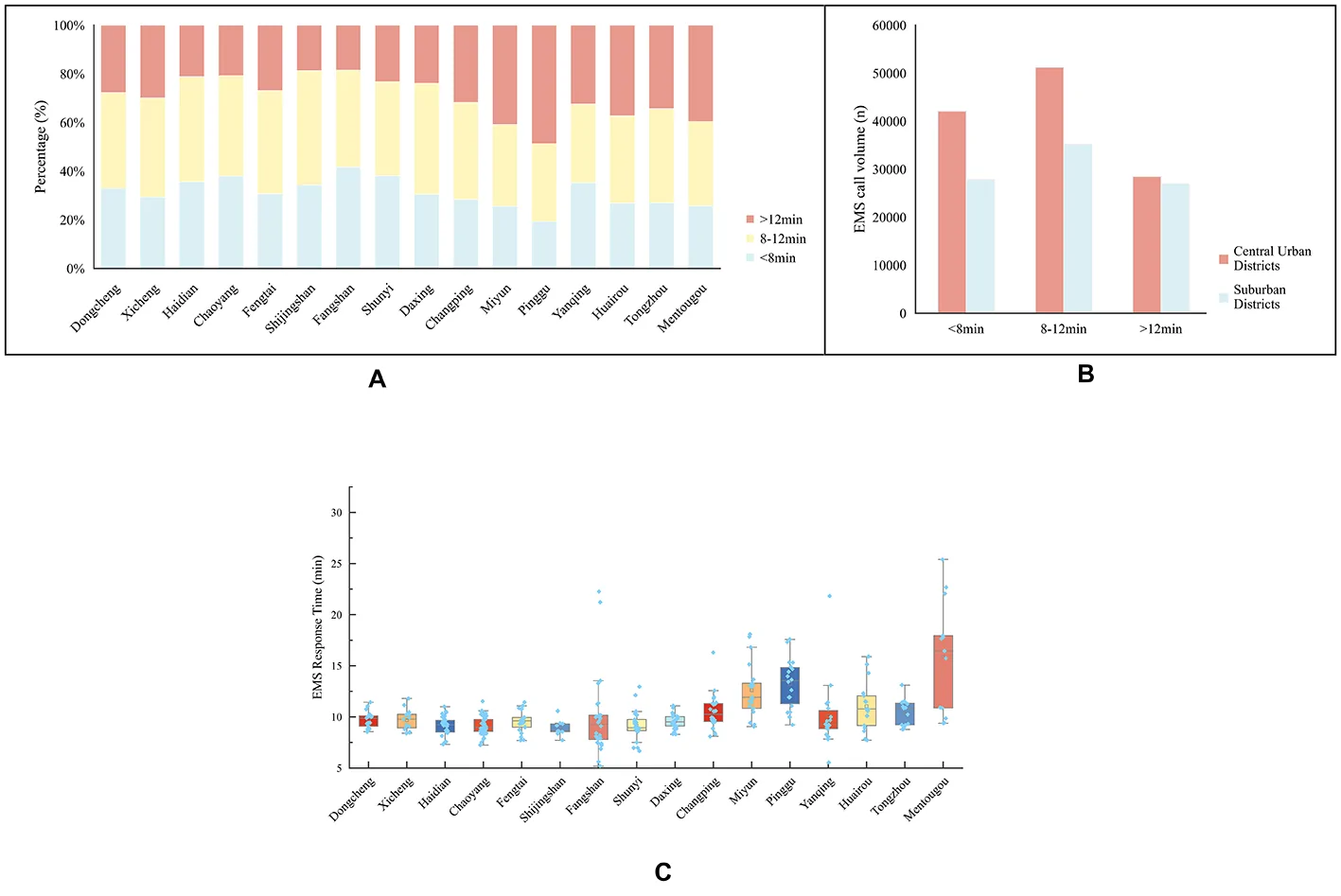
District-level variations in EMS response time in Beijing: **(A)** distribution of EMS call volumes across EMS response time based on districts; **(B)** distribution of EMS call volumes across EMS response time based on urban/suburban areas; **(C)** box plots of median EMS response time.

The distribution of EMS response time across districts is illustrated in Figure 3C. The median average EMS response time in the central urban districts, Dongcheng, Xicheng, Chaoyang, Haidian, Fengtai and Shijingshan, were generally low (all under 10 min), indicating relatively stable and less dispersed EMS response time. In contrast, the median average EMS response times in suburban districts of Pinggu (13.53 min) and Mentougou (16.45 min) were relatively higher (all exceeding 12 min) and exhibited greater variability.

### 3.2 Citywide multilevel regression results

In the full-sample model, the ICC was 0.195, indicating that approximately 19.5% of the variation in EMS response time can be attributed to differences between subdistricts. This result, together with the hierarchical structure of EMS events nested within subdistricts, justified the use of a multilevel model to account for within-subdistrict correlation. The multicollinearity diagnostics show VIF values of the control variables and all dimensional independent variables were below 10, indicating no serious multicollinearity in the model.

Table 2 presents the multilevel regression results for the full-sample model. Model 1 includes only individual-level control variables (EMS distance, peak-period dispatch, and adverse weather), while Model 2 adds subdistrict-level controls (per capita GDP, EMS call volume, EMS station density, and ambulance unit density). Models 3 incorporates the three dimensions of subdistrict-level built environment variables (density, land use, and road network). The results indicate that higher arterial road density (Coef = −25.150, *p* < 0.01) and local road density (Coef = −21.135, *p* < 0.01) were significantly associated with shorter EMS response times. Higher densities of residential POIs (Coef = 3.634, *p* < 0.05) were also associated with significantly longer EMS response time. Other built environment variables exhibited no statistically significant effects.

**Table 2:** Full-sample multilevel regression results.

Variable	Model 1		Model 2		Model 3	
	Coef.	*p*-value	Coef.	*p*-value	Coef.	*p*-value
Distance	0.090	0.000^***^	0.089	0.000^***^	0.089	0.000^***^
Peak-period dispatch	60.772	0.000^***^	57.207	0.000^***^	57.221	0.000^***^
Adverse weather	−12.025	0.000^***^	−11.708	0.000^***^	−11.714	0.000^***^
GDP per capita			−0.000	0.002^***^	0.000	0.246
EMS call volume			0.205	0.000^***^	0.205	0.000^***^
EMS station density			143.409	0.000^***^	89.268	0.000^***^
Ambulance unit density			−26.384	0.017^**^	−15.706	0.138
Population density					−0.003	0.112
Building density					−291.644	0.245
Commercial POIs density					0.003	0.978
Educational POIs density					0.118	0.910
Residential POIs density					3.634	0.035^**^
Open space ratio					−0.565	0.568
Expressway density					−16.768	0.134
Arterial road density					−25.150	0.003^***^
Collector road density					−11.765	0.223
Local road density					−21.135	0.000^***^
Intersection density					0.604	0.241
*N*	211,685					

^***^p < 0.01, ^**^p < 0.05.

### 3.3 Multilevel regression results for central urban and suburban areas

Both the central urban area model and the suburban model were estimated using multilevel models. Table 3 illustrates the differences in the effects of the built environment on EMS response time between central urban and suburban areas. In the central urban model, higher population density (Coef = −0.003, *p* < 0.01) was significantly associated with shorter EMS response time. Residential facility density, in contrast, showed a significant positive association (Coef = 1.878, *p* < 0.05), indicating that higher density may delay EMS response. Regarding the road network, higher densities of arterial roads (Coef = −12.320, *p* < 0.05) were associated with shorter response times.

**Table 3:** Multilevel regression results for central urban and suburban areas.

Variable	Central urban		Suburban areas	
	Coef.	*p*-value	Coef.	*p*-value
Population density	−0.003	0.003^***^	−0.002	0.737
Building density	−262.159	0.051^*^	259.937	0.696
Commercial POIs density	0.011	0.805	−0.221	0.450
Educational POIs density	0.025	0.960	7.291	0.582
Residential POIs density	1.878	0.024^**^	3.531	0.560
Open space ratio	−0.446	0.364	1.119	0.699
Expressway density	−5.541	0.326	3.641	0.914
Arterial road density	−12.320	0.026^**^	−18.710	0.306
Collector road density	−2.069	0.687	−40.791	0.067^*^
Local road density	−2.335	0.532	−36.509	0.001^***^
Intersection density	0.483	0.086^*^	−0.271	0.818
*N*	121,751		89,934	

^***^p < 0.01, ^**^p < 0.05, ^*^p < 0.1. The table reports only built environment variables. Both models include the same set of individual-level and subdistrict-level control variables as in the full-sample model.

In the suburban model, only local road density (Coef = −2.335, *p* < 0.01) was significantly associated with shorter EMS response time, reflecting the positive role of lower-level roads in enhancing EMS response efficiency in suburban areas.

## 4 Discussion

### 4.1 Main findings

Based on real ambulance dispatch records in Beijing and multi-source built environment data, this study constructed a multilevel multiple regression model to examine the effects of subdistrict-level built environment on EMS response time and its urban–suburban disparities. There are two main findings from this study: (1) at the citywide level, lower residential POIs density, and higher road network density (including arterial, and local roads) significantly reduce EMS response time. (2) In central urban areas, higher residential facility density may increase demand pressure and create last-mile access barriers, thereby prolonging EMS response time, while higher arterial road density improves cross-regional ambulance mobility. In contrast, in suburban areas, where residential settlements are more dispersed and road networks are less connected, only local road density showed a significant effect, with higher local road density associated with shorter EMS response time.

Our first finding reveals the relationships between built environment factors and EMS response time across the city, expanding our understanding of how the built environment can affect EMS efficiency. The results indicate that, areas with higher residential POIs density tend to have longer EMS response time. Higher residential facility density, characterized by compact building configurations and complex internal circulation systems, present substantial last-meter accessibility challenges for emergency responders at the micro scale (61). At the macro scale, such areas are frequently associated with intensive pedestrian-vehicle interactions and road congestion, further compromising the travel efficiency of EMS vehicles. In addition, our study shows that higher road network density across different levels is significantly associated with shorter EMS response time. Specifically, higher arterial road density is associated with shorter response times (21). These roads typically feature higher design speeds and standardized intersection controls, providing continuous and efficient travel conditions for ambulances (62). Simultaneously, robust network connectivity may alleviate congestion and improve overall travel efficiency (15). A high-density network of local roads provides ambulances with more flexible route choices at the micro-level, avoiding delays caused by overly large block sizes or monotonous road structures (63). China's recent advocacy for the “narrow roads, dense networks” urban design strategy aims precisely to enhance traffic conditions by increasing road network density and accessibility, thereby further improving the efficiency of various emergency services, including the EMS.

Our second finding underscores the significance of spatial location in understanding the relationship between the built environment and EMS response time. In central urban areas, high-density residential facilities are linked to longer response times, whereas this effect is not significant in suburban areas. A likely explanation is that higher residential facility density impedes EMS accessibility and travel efficiency due to complex building layouts, intensive pedestrian–vehicle interactions, and traffic congestion (64, 65). Notably, in the central urban areas, population density was negatively associated with EMS response time. This finding suggests that residential facility density and population density may capture different aspects of the urban environment. In central urban areas, higher residential facility density may reflect compact building configurations and complex internal circulation systems. These areas often have narrow internal roads, restricted entrances, on-street parking, and complex internal traffic organization, which may increase the “last-mile” delay for ambulances to reach the patient location (38). In contrast, higher population density may correspond to mature large-scale residential communities, better public services, and higher levels of EMS resource allocation, which may have advantages in service coverage, ambulance dispatch, and infrastructure accessibility, leading to shorter EMS response times (16). Therefore, the results indicate that population density and residential facility density may affect EMS response efficiency through different mechanisms in central urban areas.

The impact of the road network on EMS response time also exhibits urban–suburban differences. High-density arterial roads in central urban areas can significantly improve EMS efficiency, but this effect is not pronounced in suburban regions. A possible reason is that arterial roads in urban cores serve as the primary corridors for rapid cross-district movement. EMS vehicles rely on road systems with high continuity, connectivity, and traffic capacity to minimize travel time over longer distances. Therefore, a higher density of arterial roads provides greater benefits for emergency response efficiency in central urban areas. By contrast, suburban areas typically feature hierarchical tree-like road networks with a higher proportion of lower-level roads, limiting the accessibility and traffic flow advantages of arterial roads (66). In contrast, increasing the density of local roads can reduce EMS response time, particularly in suburban areas. In these settings, the key challenge is often not rapid long-distance travel but access to dispersed villages, residential communities, and industrial parks. A denser local road network improves spatial coverage and enhances the “last-mile” accessibility of EMS services, enabling ambulances to reach incident locations more directly (67). In central urban areas, however, the effectiveness of local roads is constrained by traffic congestion, on-street parking, and other operational disruptions, which reduce the flexibility and efficiency of lower-level road networks (68). These findings suggest that different road hierarchies contribute to EMS performance through distinct mechanisms: arterial roads primarily support rapid regional movement, whereas local roads enhance fine-grained accessibility and spatial coverage.

### 4.2 Strengths and limitations

This study has three key strengths. First, it uses Beijing, a typical high-density megacity, as the study area. This extends previous research on the built environment and emergency medical service (EMS) response time, which has mainly focused on low-density cities in Europe and North America, and provides evidence on how the built environment is associated with EMS response time in high-density urban settings. Second, the study shows that the associations of the built environment on EMS response time vary between urban and suburban areas. The differences are mainly reflected in the varying strength and direction of the effects of road hierarchy and facility clustering and support the development of differentiated urban–suburban policies. Third, the study employs a subdistrict–individual multilevel regression model, which overcomes the limitations of traditional single-level analysis and enables a more rigorous and accurate assessment of how the built environment influences EMS response time.

Several questions are worthy of further exploration. First, the relationship between the built environment and EMS response time may involve non-linear or threshold effects (69). Future research could jointly consider nested data structures and non-linear relationships. Second, all road network indicators were density-based and primarily reflected road infrastructure availability. Future studies should incorporate network topology measures to better characterize road network performance. Third, this study analyzed the overall response time from call receipt to scene arrival. This aggregation may obscure stage-specific associations with the built environment. Therefore, future research could further investigate the EMS response process by collecting and analyzing data for its individual sub-stages. Finally, the lack of ambulance GPS trajectory data limited our ability to accurately capture the built environment along actual travel routes. Straight-line EMS distance may also fail to reflect route complexity, while dynamic traffic conditions, such as congestion and ambulance speed, were not included. Future studies could incorporate GPS trajectory data to improve the measurement of travel routes, built environment exposure, and dynamic traffic conditions.

## 5 Conclusions

This study investigated the associations between subdistrict-level built environment characteristics and EMS response time and its urban–suburban disparities by utilizing 2024 ambulance dispatch records of Beijing with multi-source built environment data and a multilevel regression model. Results indicate that higher densities of residential facilities in central urban areas were associated with longer EMS response time. In contrast, higher population density and arterial road density in central urban areas, as well as higher local road density in suburban areas, were associated with greater EMS efficiency.

These findings highlight several potential directions for EMS planning and urban management. First, ambulance units and medical staff could be allocated more efficiently according to population distribution and historical EMS demand, particularly in high-demand areas. Second, in densely developed residential areas, better management of roadside parking, emergency entrances, and internal roads may help improve ambulance access. Finally, closer coordination between EMS and transportation authorities, together with the integration of real-time traffic information into dispatch systems, could support route selection and operational efficiency.

## Data Availability

The datasets presented in this article are not readily available because the original data is the desensitization data provided by the Beijing Emergency Medical Center and is not available for public disclosure. Requests to access the datasets should be directed to Jingwei Li, jingweili2019@126.com.
